# Dr. William E. Palmer

**Published:** 1905-01

**Authors:** 


Dr. William E. Palmer, of Spokane, Wash., a graduate of the University of Buffalo in 1897, died November 21, 1904, aged 32 years. His death resulted from acute nephritis following an operation for appendicitis.

